# Tn3-derived inverted-repeat miniature elements that mobilize antibiotic resistance genes

**DOI:** 10.1099/mgen.0.001790

**Published:** 2026-07-14

**Authors:** Ryota Gomi, Hirokazu Yano

**Affiliations:** 1 Research Center for Water Environment Technology, School of Engineering, The University of Tokyo, Bunkyo-ku, Tokyo, Japan; 2 Department of Environmental Engineering, Graduate School of Engineering, Kyoto University, Katsura, Nishikyo-ku, Kyoto, Japan; 3 Antimicrobial Resistance Research Center, National Institute of Infectious Diseases, Japan Institute for Health Security, Higashimurayama, Tokyo, Japan

**Keywords:** antibiotic resistance, *Enterobacteriaceae*, miniature inverted-repeat transposable element, mobile genetic element

## Abstract

Miniature inverted-repeat transposable elements (MITEs) are non-autonomous mobile genetic elements (MGEs) that can be mobilized by transposases provided by the relevant autonomous MGEs. MITEs originating from Tn*3*-family transposons were previously termed Tn*3*-derived inverted-repeat miniature elements (TIMEs). Composite transposon-like structures bounded by two copies of TIME, called TIME-COMPs, were shown to mobilize the intervening sequences. However, their association with antibiotic resistance genes (ARGs) has not yet been systematically studied. This study thus aimed to identify new TIME-COMP-like structures containing ARGs in the genomic sequences of the clinically important bacterial family *Enterobacteriaceae* in public databases. TIME-COMP-like structures were first searched for in the plasmid database PLSDB, focusing on small plasmids, using a self-against-self blastn approach to identify repeated elements. Then, newly and previously identified MITEs (including TIMEs) were searched for in the NCBI core nucleotide database to identify TIME-COMP-like structures located on other replicons. Bioinformatic analysis identified multiple previously unreported TIME-COMPs containing ARGs, which are bounded by directly or inversely oriented TIMEs, namely IS*101*, MITESen1 and a novel 244 bp TIME termed TIME244. TIME244 contains a putative resolution site related to that of Tn*21*. These TIMEs were predominantly detected in plasmids and very rarely in chromosomes. The ARGs embedded in newly identified TIME-COMPs were *bla*_KPC-2_, *floR*, *qnrS1* and *tet*(A). Notably, the *bla*_KPC-2_ carbapenemase gene was found in TIME-COMPs bounded by TIME244 and a TIME-COMP bounded by IS*101*. These findings highlight a potential role for TIMEs in the spread of diverse ARGs.

**Impact Statement**

Bacterial miniature inverted-repeat transposable elements (MITEs) are a group of short (50–500 bp) non-autonomous transposable elements that are thought to have originated from insertion sequences or transposons. Although MITEs can theoretically mobilize antibiotic resistance genes (ARGs) in the presence of transposases, only a few studies have reported their association with ARGs, probably due to difficulties in identifying MITEs in genomic sequences. This study provides evidence, based on bioinformatic analysis of public *Enterobacteriaceae* genomes, that a subset of MITEs, called Tn*3*-derived inverted-repeat miniature elements (TIMEs), mobilizes ARGs by forming composite transposon-like structures. A novel 244 bp TIME, designated TIME244, was present in more than 100 *Enterobacteriaceae* plasmids in the current RefSeq database, suggesting its further transmission in bacterial populations through horizontal gene transfer. This study reveals that TIMEs were often overlooked when analysing the genetic contexts of ARGs in previous studies. These findings highlight the importance of TIMEs in bacterial gene acquisition and underscore the need for new tools that can detect TIMEs in bacterial genomes for ARG surveillance.

## Data Summary

Accession numbers of sequence data analysed in this study are provided within the article or in supplementary data files.

## Introduction

Antibiotic-resistant bacteria, particularly those with multidrug resistance (MDR), are a global health concern. Bacteria can accumulate antibiotic resistance genes (ARGs) with the help of mobile genetic elements (MGEs), which can often lead to MDR. MGEs can be categorized into two types: those mobilizing ARGs from one cell to another cell (i.e. intercellular mobilization) and those mobilizing ARGs from one replicon to another replicon or to different locations within the same replicon (i.e. intracellular mobilization) [1]. Intercellular mobilization mechanisms such as conjugation can directly contribute to horizontal transfer of ARGs and thus the emergence and spread of MDR. Intracellular mobilization can also contribute to the increase of MDR in multiple ways. For example, it can lead to the movement of ARGs from the chromosome to a plasmid or from a plasmid to different plasmids, thereby increasing the potential of the ARGs to be transmitted to other bacterial recipients via conjugation [2]. Additionally, it enables the accumulation of ARGs in a single genomic location to form a multiresistance region [3], facilitating their co-transfer as a single unit.

Transposable elements (TEs) can facilitate the intracellular mobilization of ARGs. TEs frequently associated with ARGs include insertion sequences (ISs), IS-composite transposons and unit transposons such as the Tn*3* family [1]. The majority of these elements encode DDE transposases [4]. DDE transposases catalyse reactions such as the excision of a double-stranded TE bounded by inverted repeats (IRs) from its flanking DNA (for example, Tn*5*) or the nicking of the 3′ ends of a TE (for example, Tn*3*), followed by the transfer of the two 3′-OH ends to the two strands of a target site. Subsequent gap filling of the resulting strand-transfer product generates direct repeats (DRs) that flank the newly inserted TE, serving as a hallmark of the transposition event [4]. A subset of TEs, called non-autonomous elements, can also mediate intracellular mobilization but require the transposase activity provided in *trans* by autonomous elements [1]. Among the known non-autonomous elements are structures called miniature inverted-repeat transposable elements (MITEs) [5]. MITEs are thought to have originated from ISs or transposons. They retain the terminal IRs but lack the internal regions, including the transposase gene(s). They are typically 50–500 bp and also create DRs upon insertion [6].

A subset of MITEs originating from Tn*3*-family transposons and carrying Tn*3*-related IRs are called Tn*3*-derived inverted-repeat miniature elements (TIMEs), and they include elements such as IS*101*, integron mobilization unit (IMU), MITESen1 and TIME1–TIME4 [7,10]. Composite transposon-like structures bounded by two copies of TIME, known as TIME-COMPs, were shown to mobilize the intervening sequences when the transposase activity of autonomous Tn*3*-family elements was provided in *trans* [7,9]. However, to date, known TIME-COMPs carrying an ARG are limited to IMU-bounded TIME-COMPs carrying a carbapenemase gene *bla*_GES-5_ and an IS*101*-bounded TIME-COMP carrying a quinolone resistance gene *qnrD1*, even in the clinically important bacterial family *Enterobacteriaceae* (Fig. S1, available in the online Supplementary Material) [9,11, 12]. MITEs other than TIMEs should also be capable of mobilizing an ARG when the ARG is embedded within a TIME-COMP-like structure; however, to our knowledge, no structures bounded by non-TIME MITEs have been reported in *Enterobacteriaceae*.

Tn*3*-family transposons have been identified in nearly all bacterial phyla and are classified into several subgroups (including Tn*3* and Tn*21*) based on the phylogeny of their transposases [13]. These elements mobilize through a ‘paste-and-copy’ mechanism involving two distinct steps: cointegrate formation and cointegrate resolution. The first step is mediated by the transposase (the *tnpA* gene product), IRs and the host replication machinery, generating a fusion replicon in which the donor and target replicons are bridged by directly repeated copies of the transposon. In the second step, the cointegrate is resolved into two separate molecules, each carrying a transposon insertion. This resolution is typically mediated by the transposon-encoded resolvase (the *tnpR* gene product) and its cognate target site (*res*) or through homologous recombination between the duplicated transposon sequences. TIME and TIME-COMP elements are hypothesized to employ a similar ‘paste-and-copy’ mechanism; however, whether these elements contain functional resolution sites remains unclear in most cases, with the exception of IS*101*, which possesses a functional *res* site [14].

Transposition of an IMU-bounded TIME-COMP was previously demonstrated by providing a transposase of IS*Sod9* belonging to the Tn*21* subgroup [9]. IS*101* cointegrate formation and resolution were demonstrated in the presence of Tn*1000* (also called gamma-delta), belonging to the Tn*3* subgroup [14]. Known TIMEs and TIME-COMPs create 5 bp (or sometimes 6 bp) DRs upon transposition like Tn*3*-family transposons [7,9, 13]. Thus, past transposition of a TIME-COMP can be inferred from the presence of 5 bp or 6 bp DRs flanking the outermost IRs of the TIME-COMP.

MITEs (including TIMEs) tend to be overlooked in standard annotation pipelines due to their lack of transposase gene(s). Furthermore, as of January 2026, the number of MITE sequences registered in the ISFinder database remained at 65, with only three originating from *Enterobacteriaceae* [15]. Thus, the contribution of MITEs to the intracellular mobility of ARGs is likely underestimated. Hence, this study aimed to identify previously overlooked TIME-COMP-like structures carrying ARGs in *Enterobacteriaceae* by data mining.

## Methods

### Plasmid sequences

Plasmid sequences (*n*=72,556) were downloaded from PLSDB (v. 2024_05_31_v2), which is a curated and non-redundant plasmid database sourced from the National Center for Biotechnology Information (NCBI) database [16]. Plasmid sequences meeting the following criteria were extracted: (i) at least one ARG was detected by ABRicate (v1.0.1, https://github.com/tseemann/abricate) with the NCBI database [17]; (ii) at least one plasmid replicon was detected by ABRicate (v1.0.1) with the ‘--db plasmidfinder’ option [18] (this was to exclude sequences that are not likely to be true plasmids and to be conservative; for example, NZ_CP107371 is composed almost entirely of ARGs and ISs/transposons, suggesting a non-plasmid circular element or a misassembled circular contig); (iii) the plasmid host is *Enterobacteriaceae*; and (iv) the length is 20,000 bp or shorter. Here, we focused on small plasmids because their small size allows manual curation of the results of bioinformatic analysis (described in detail below). Plasmid sequences meeting the above criteria (*n*=1,007) were subjected to self-against-self blastn analysis as described below to detect repeated sequences that could be MITEs.

### Detection of TIME-COMP-like structures carrying ARGs

The blast+ (v2.16.0) program was used for similarity searches. The makeblastdb and blastn functions were used with default parameters. Plasmid sequences with self-against-self blastn hits meeting the following criteria were retained: (i) the alignment length is between 50 bp and 500 bp; (ii) the percentage of identical matches is 98 % or higher; and (iii) a sequence longer than 500 bp is present between the aligned part of the query sequence and that of the subject sequence. These criteria were applied to detect repeat sequences (50–500 bp) surrounding a stretch of sequence (>500 bp) in a plasmid (see Fig. S2 for the rationale for these criteria). To confirm that the 50-bp to 500-bp repeat sequences are actually MITEs, a self-against-self blastn search was performed for each repeat sequence to identify IRs, which are the signature of MITEs. The blastn search was performed by using a word size of six to increase the sensitivity, and the result was visualized using CLC Main Workbench 24 (QIAGEN, Hilden, Germany) (Fig. S3). The putative MITE sequences were checked for identity to any known elements in the ISfinder database [15]. Only TIME-COMP-like structures flanked by DRs, which are typically created upon insertion of the entire structure, were considered for further analysis. This was to exclude cases where two MITEs were independently inserted and coincidentally formed a TIME-COMP-like structure. The lengths of DRs looked for were determined with reference to those of the related ISs or transposons. Plasmids with TIME-COMP-like structures carrying ARGs were annotated using ISfinder and the blastn web tool (https://blast.ncbi.nlm.nih.gov/Blast.cgi).

The MITE(s) found in the above approach, previously described TIMEs associated with ARGs in *Enterobacteriaceae* (i.e. IMU and IS*101*) [9,11, 12], and *Enterobacteriaceae* MITEs listed in the ISfinder database (i.e. MITEKpn1 and MITESen1, excluding MITEEc1, which is an enterobacterial repetitive intergenic consensus sequence and present in multiple copies on the chromosome of *Escherichia coli*) [15,19, 20], were searched for in the NCBI core nucleotide database (core_nt) using the blastn web tool (last accessed July 2025). Replicons with two or more blastn hits with ≥95 % identity and ≥95 % coverage were manually screened to detect additional TIME-COMP-like structures carrying an ARG in *Enterobacteriaceae*. This enabled identification of TIME-COMP-like structures in small plasmids not indexed in PLSDB, in longer plasmids and also in chromosomes. (Note that we also performed the online blastn analysis described above for TIME1–TIME4, which were originally described in *Pseudomonas* spp. [7], and confirmed the absence of these elements in *Enterobacteriaceae*.)

Putative ancestral plasmids without insertion of TIME-COMP-like structures were identified by performing the online blastn analysis against the NCBI core_nt database, using a sequence, which was prepared by joining the sequences flanking the TIME-COMP-like structure minus one copy of the DR, as a query sequence (last accessed January 2026).

Sequence similarity among putative *res* regions of Tn*3*-family elements was evaluated using the SSEARCH program (ssearch36) from the FASTA package v.36.3.8i with default parameters [21,22], querying a multi-FASTA file of putative *res* regions against itself. Smith–Waterman scores (S-W) above 100 were considered significant matches.

### Detection of MITEs in RefSeq *Enterobacteriaceae* genomes

To investigate the prevalence of MITEs that were found to be associated with ARGs, we downloaded RefSeq *Enterobacteriaceae* genomes with an assembly level of ‘complete genome’ using the NCBI Datasets command-line tools (v18.9.0) in October 2025 (*n*=11,572) [23]. MITE sequences were searched for in the retrieved genomes using makeblastdb and blastn in blast+ (v2.16.0) with default parameters. We used the same criteria as the online blastn search to define the presence of MITE sequences (i.e. ≥95 % identity and ≥95% coverage).

## Results and discussion

### Identification of TIME-COMP-like structures carrying ARGs

First, we conducted self-against-self blastn analysis to identify TIME-COMP-like structures in a subset of small *Enterobacteriaceae* plasmids registered in the curated plasmid database, PLSDB. This relatively small dataset (*n*=1,007) made manual curation of the blastn results feasible, which includes confirmation of the IRs in putative MITEs and DRs flanking the entire TIME-COMP-like structures. These procedures revealed TIME-COMP-like structures carrying ARGs in six plasmids (Table S1). All these structures are bounded by TIMEs and thus are TIME-COMPs. One of these plasmids is pKFu015_4, which was previously reported to carry the IS*101*-bounded TIME-COMP containing *qnrD1* (Fig. S1) [12]. The remaining five plasmids contain the same TIME-COMP structure with *bla*_KPC-2_ and a truncated *bla*_TEM_ gene, which is bounded by two copies of a 244 bp TIME. This element was submitted to ISfinder and designated as MITEEc2 [15]; however, TIME244 was also registered as a synonym, which is used in this study to explicitly highlight that this element is indeed a TIME. pCHE-A and pCHE-A1, which were previously reported to contain IMU-bounded TIME-COMPs (Fig. S1), were detected by the self-against-self blastn searches but were filtered out in manual curation, because the TIME-COMP structures in these plasmids are not flanked by DRs [9,11]. We also detected six other plasmids carrying TIME-COMP structures with ARGs but not flanked by DRs, all bounded by MITESen1 (see Fig. S4 for details). These TIME-COMP structures may have the potential to transpose as a unit; however, these cases were filtered out to restrict the dataset to plasmids showing direct evidence of transposition.

To identify additional TIME-COMP-like structures in replicons other than small plasmids indexed in PLSDB, we performed online blastn searches using TIME244 and previously reported MITEs, including those classified as TIMEs (IS*101*, IMU and MITESen1) and one non-TIME MITE (MITEKpn1), as query sequences (see Table S2 for the sequence of each MITE). This online blastn analysis identified 141 additional replicons carrying TIME-COMPs with ARGs. These included 136 plasmids and 1 chromosome carrying TIME244-bounded TIME-COMPs [most of which contain *tet*(A)], 1 plasmid carrying an IS*101*-bounded TIME-COMP and 3 plasmids carrying MITESen1-bounded TIME-COMPs (Table S1).

The genetic contexts of TIME-COMPs identified using the above approaches were thoroughly inspected as described below.

### TIME244-bounded TIME-COMPs

As mentioned above, we detected TIME244-bounded TIME-COMPs in a total of 142 replicons (i.e. 5 identified by the self-against-self blastn analysis and 137 identified by the online blastn analysis). Three main types of TIME244-bounded TIME-COMPs were identified.

**Fig. 1.**
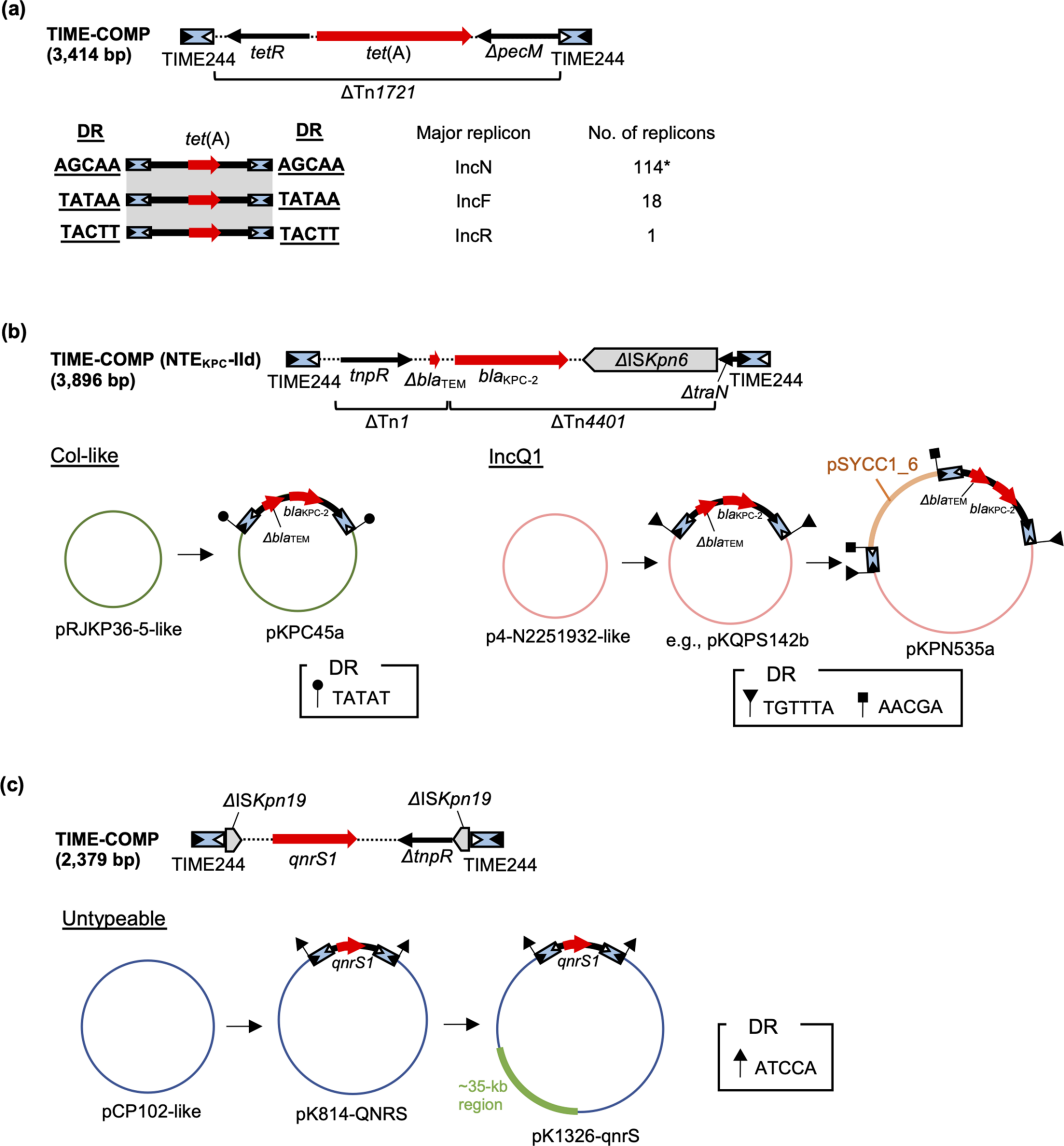
(**a**) TIME244-bounded TIME-COMP carrying *tet*(A) and DRs flanking the TIME-COMP. The expanded diagram of the TIME-COMP is shown at the top. Major replicon types and the number of replicons are shown on the right side. *There are variants in TIME-COMPs flanked by AGCAA (see Table S1 for details). (**b**) TIME244-bounded TIME-COMP carrying *bla*_KPC-2_ and a truncated *bla*_TEM_ gene and plasmids carrying the TIME-COMP. The expanded diagram of the TIME-COMP is shown above the plasmids. For the TIME-COMP structure in each plasmid, only TIMEs and ARGs are depicted for clarity. The TIME-COMP was inserted into a pRJKP36-5-like plasmid and a p4-N2251932-like plasmid, leading to pKPC45a and pKQPS142b (and four other IncQ plasmids showing >99% identity and >99% coverage against pKQPS142b, as in Table S1), respectively. In pKPN535a, three copies of TIME244 are present, and a sequence identical to pSYCC1_6 is present between two of the TIME244 copies. This indicates a cointegrate formation by ‘paste-and-copy’ transposition of TIME244, leading to fusion of a pKQPS142b-like plasmid and pSYCC1_6. The online blastn analysis identified eight putative ancestral plasmids (including pRJKP36-5) for pKPC45 and seven putative ancestral plasmids (including p4-N2251932) for pKQPS142b, showing >90% identity and >98% coverage against the uninterrupted version of each plasmid. (**c**) TIME244-bounded TIME-COMP carrying *qnrS1* and plasmids carrying the TIME-COMP. The TIME-COMP was likely inserted in a pCP102-like plasmid, leading to pK814-QNRS. pK814-QNRS then acquired a ~35 kb region with high similarity to the partial sequence of *Klebsiella* phage KP12 clone KP12_2 (OM835952) (96% identity and 93% coverage), leading to pK1326-qnrS. The online blastn analysis identified three putative ancestral plasmids (including pCP102) for pK814-QNRS, showing >90% identity and >80% coverage against the uninterrupted version of pK814-QNRS. Red arrows indicate ARGs, grey pointed boxes indicate ISs, blue boxes indicate TIMEs and black arrows indicate other genes. The IRs within each TIME are shown by a black (left, IRL) or white (right, IRR) triangle (see Table S2 for the basis of the orientations of TIMEs). DRs are shown as differently shaped flags, and their sequences are shown in boxes. The accession numbers of the plasmids are as follows: pRJKP36-5, CP100081; pKPC45a, MH595534; p4-N2251932, CP165866; pKQPS142b, CP023480; pKPN535a, MH595533; pSYCC1_6, CP113183; pCP102, CP104015; pK814-QNRS, CP183858; and pK1326-qnrS, CP183864.

One of them is a TIME244-bounded TIME-COMP carrying *tet*(A) detected in 133 replicons (Table S1). This TIME-COMP is bounded by inversely oriented TIME244 copies and contains part of Tn*1721*, an 11 kb Tn*3*-family element carrying tetracycline resistance determinant genes originally identified on an *E. coli* plasmid (Fig. 1a) [24,25]. This TIME-COMP was inserted in three unique locations with DRs of AGCAA (*n*=114), TATAA (*n*=18) and TACTT (*n*=1) (Fig. 1a and Table S1). This indicates that three independent transposition events of this TIME-COMP had occurred in the past. In 114 replicons with DRs of AGCAA, 2 types of the TIME-COMP were identified: one with an inverted internal structure (*n*=52) and the other without (*n*=62) (see below for detailed discussion). Furthermore, there were several indels within the TIME-COMP structure, two of which involved alterations of ARG contents (Fig. S5; see Table S1 for detailed description). Briefly, in a plasmid, a resistance region containing *bla*_TEM-1B_ and *bla*_CTX-M-206_ is inserted within the left TIME244 copy. In the other plasmid, the Tn*1721*-derived region is partially deleted and replaced by a truncated Tn*3*-like transposon and a truncated Tn*4401*-like transposon containing *bla*_KPC-2_ (i.e. a variant of non-Tn*4401* elements (NTE_KPC_), which contains only a portion of Tn*4401*, the most common *bla*_KPC_-containing mobile element belonging to the Tn*3* family) [26]. In these cases, the DR sequences flanking the TIME-COMPs are the common ones (AGCAA); thus, the indels were likely introduced after the insertion of the original TIME-COMP carrying *tet*(A). The outermost IRs within the altered TIME-COMP structures are intact, implying the feasibility of further transposition if the relevant transposase activity is provided in *trans* (Fig. S5).

Another TIME-COMP carries *bla*_KPC-2_ and a truncated *bla*_TEM_ gene, which is bounded by directly oriented TIME244 copies (Fig. 1b). This genetic context was previously described as a variant of NTE_KPC_, designated NTE_KPC_-IId [27]. Although the study noted repeats at its boundaries, the size was described as 243 bp rather than 244 bp, likely because the Tn*3*-related IRs were not fully recognized. Furthermore, these repeats were not identified as MITEs or TIMEs. This TIME-COMP was inserted in the same site in six highly related IncQ1 plasmids, with the same DRs (TGTTTA), and in a single Col-like plasmid with DRs of TATAT (Fig. 1b and Table S1). In one of the IncQ1 plasmids (pKPN535a), three copies of TIME244 are present, which could be explained by the fusion of two plasmids.

We also detected another TIME-COMP, which contains *qnrS1* and is bounded by inversely oriented TIME244 copies, in two untypeable plasmids (Fig. 1c) [28,29]. In these plasmids, the TIME-COMP structure is inserted in the same position with DRs of ATCCA; therefore, a total of one unique transposition event was detected. These two plasmids and their putative ancestral plasmid were classified as phages by geNomad (v1.11.2) [30]. Thus, these are putative phage-plasmids, which transfer horizontally between cells as viruses and vertically within cellular lineages as plasmids [31].

### An IS*101*-bounded TIME-COMP

**Fig. 2.**
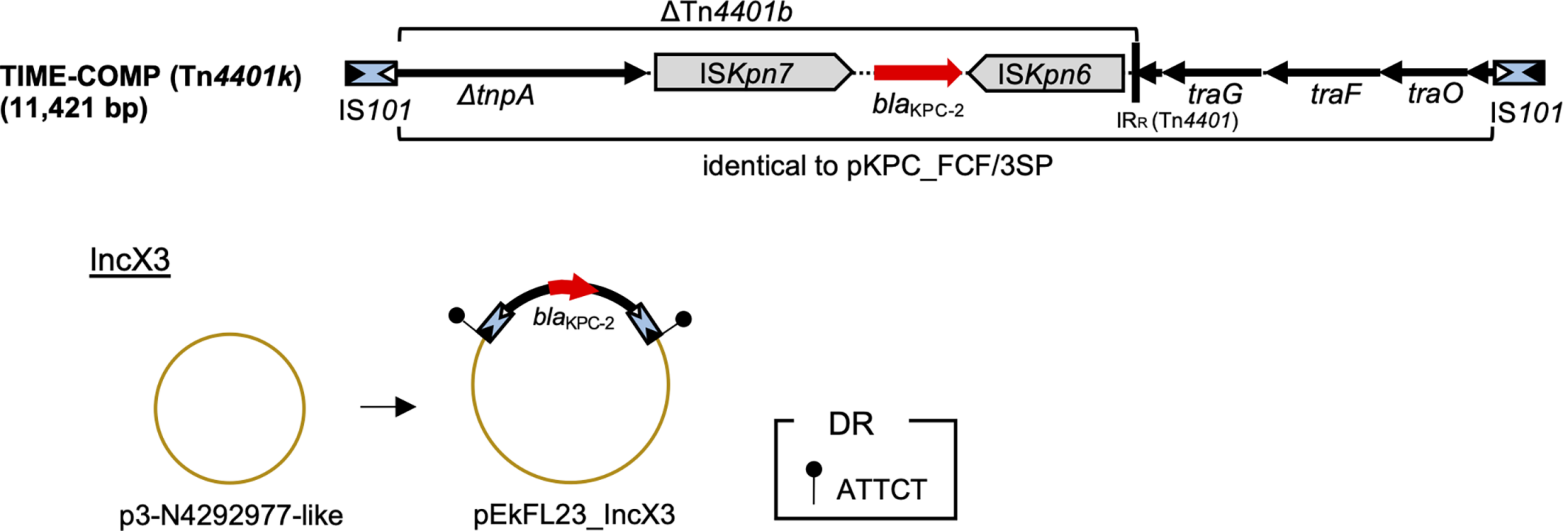
IS*101*-bounded TIME-COMP carrying *bla*_KPC-2_ and a plasmid carrying the TIME-COMP. The TIME-COMP was inserted into a p3-N4292977-like plasmid, leading to pEkFL23_IncX3. The online blastn analysis identified 19 putative ancestral plasmids (including p3-N4292977) for pEkFL23_IncX3, showing >99% identity and >90% coverage against the uninterrupted version of pEkFL23_IncX3. Genes and elements are shown as in Fig. 1. The accession numbers of the plasmids are as follows: pKPC_FCF/3SP, CP004367; p3-N4292977, CP165819; and pEkFL23_IncX3, OQ434669.

IS*101*, a 209 bp element, is among the earliest identified TIMEs and was originally discovered on a recombinant *E. coli* plasmid, pSC101 [8]. We identified a previously unreported IS*101*-bounded TIME-COMP in one *Enterobacter kobei* IncX3 plasmid, pEkFL23_IncX3 (Fig. 2). This TIME-COMP is bounded by inversely oriented copies of IS*101* and carries *bla*_KPC-2_ embedded within a truncated Tn*4401b* structure [32]. This genetic context was previously reported as Tn*4401k*, but the presence of IS*101* was not addressed in the previous study [33]. The sequence between the two IS*101* copies in this TIME-COMP corresponds to the partial sequence of plasmids such as pKPC_FCF/3SP, and the whole TIME-COMP structure was flanked by 5 bp DRs (ATTCT). These indicate that IS*101*-mediated transposition of part of a pKPC_FCF/3SP-like plasmid into another plasmid generated pEkFL23_IncX3.

### MITESen1-bounded TIME-COMPs

**Fig. 3.**
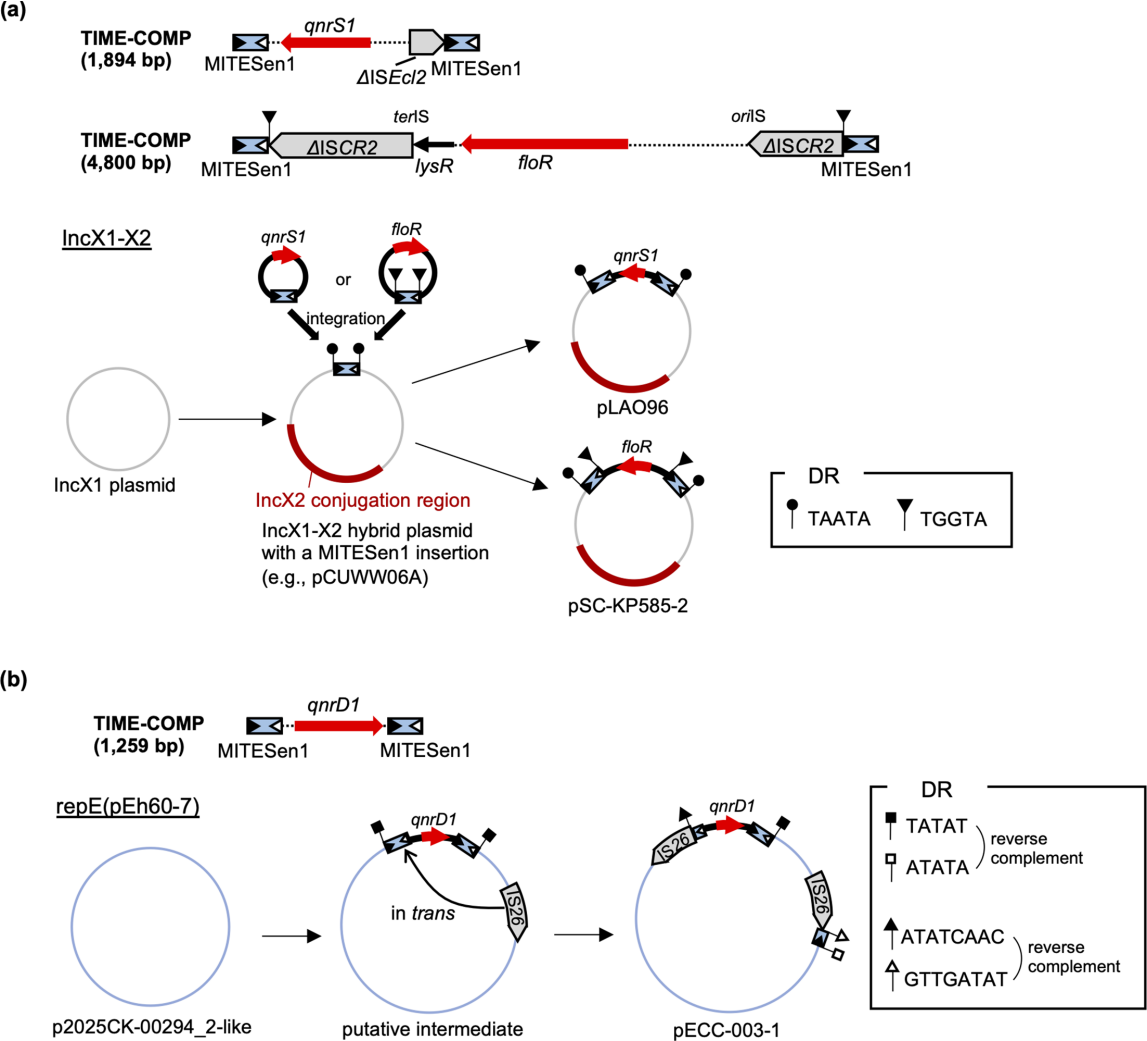
(**a**) MITESen1-bounded TIME-COMPs carrying *qnrS1* and *floR* and plasmids carrying these TIME-COMPs. pLAO96 and pSC-KP585-2 might have been generated by integration of a hypothetical circular molecule into a pCUWW06A-like plasmid. pLAO96 (45,300 bp) and pSC-KP585-2 (44,092 bp) share ~35.6 kb of highly similar sequence (>99% identity), excluding the TIME-COMP regions (1,894 bp and 4,800 bp) and regions bounded by IS*26* (7,824 bp and 3,609 bp). (**b**) MITESen1-bounded TIME-COMP carrying *qnrD1* and a plasmid carrying the interrupted TIME-COMP. A putative intermediate plasmid carrying the intact TIME-COMP is also shown. ‘in *trans*’ indicates intramolecular replicative transposition (in *trans*) of IS*26*. The online blastn analysis identified one putative ancestral plasmid (p2025CK-00294_2) for pECC-003-1, showing >99% identity and 86% coverage against the uninterrupted version of the putative intermediate. Genes and elements are shown as in the previous figures. The accession numbers of the plasmids are as follows: pCUWW06A, CP182140; pLAO96, OP242301; pSC-KP585-2, CP123874; p2025CK-00294_2, CP194151; and pECC-003-1, CP143772.

MITESen1 is a 256 bp TIME first identified on an IncX1 plasmid from *Salmonella enterica* [10]. We identified two different MITESen1-bounded TIME-COMPs in the same location with the same flanking 5 bp DRs (TAATA) in two IncX1-IncX2 hybrid plasmids (Fig. 3a) [34,35]. One TIME-COMP contains *qnrS1*, and the other contains *floR*. MITESen1 copies are directly oriented in these TIME-COMPs. Interestingly, there are IncX1-X2 hybrid plasmids carrying a single MITESen1 copy inserted in the same location. We speculate that the two plasmids with TIME-COMPs were generated by integration of a hypothetical circular molecule containing MITESen1 and an ARG into a plasmid already carrying a single copy of MITESen1. The hypothetical circular molecules might be generated by recombination between MITESen1 in a TIME-COMP or by intramolecular replicative transposition of MITESen1 (also see Fig. S6 for another possible pathway for the generation of the *floR*-containing circular element). Integration of the circular molecule might occur by homologous recombination between MITESen1 copies.

A plasmid with the repE(pEh60-7) replicon (pECC-003–1) was found to carry a remnant of a MITESen1-bounded TIME-COMP carrying *qnrD1* (Fig. 3b) [36]. One copy of MITESen1 is interrupted by IS*26*, while the truncated remnant of MITESen1 is present next to another copy of IS*26* that is oriented in the opposite direction. This arrangement is consistent with intramolecular replicative transposition (in *trans*) of an IS*26* element into MITESen1 in the intact TIME-COMP structure [37]. The presence of 8 bp DRs (ATATCAAC or its reverse complement) next to each IS*26* element on the MITESen1-sides supports this, though the intact version of this TIME-COMP structure was not present in the NCBI core_nt database.

### Overview of TIME-COMP characteristics

Table 1 summarizes the TIME-COMPs identified in this study as well as those reported previously. Four types of TIMEs (i.e. TIME244, IS*101*, MITESen1 and IMU) form TIME-COMPs with ARGs. Seven different intact ARGs were identified in these TIME-COMPs, including the carbapenemase genes *bla*_KPC-2_ and *bla*_GES-5_. Interestingly, the same TIMEs are associated with different ARGs, and the same ARGs are associated with different TIMEs. For example, TIME244 is associated with *tet*(A), *bla*_KPC-2_ (and Δ*bla*_TEM_) and *qnrS1*, while *bla*_KPC-2_ and *qnrS1* are also associated with IS*101* and MITESen1, respectively. The TIME-COMPs exhibit a broad size distribution, spanning from slightly above 1 kbp to over 10 kbp, implying that TIMEs can mediate the transposition of relatively long DNA segments. The replicon types carrying these TIME-COMPs are diverse; however, a distinct correspondence is observed between specific DR sequences and replicon types. This supports the idea that a TIME-COMP was inserted into an ancestor of that replicon type in a single event, followed by minor rearrangements that yielded the currently detected variants. However, in some cases for the TIME-COMP with *tet*(A), the TIME-COMP is carried by replicons different from the major replicon types (Table S1), indicating that MGEs other than TIMEs transposed a larger segment containing the TIME-COMP to other types of replicons. Of particular note is the overwhelming dominance of the TIME-COMP carrying *tet*(A), which may be attributed to two main reasons. One is the structural stability of the TIME-COMP bounded by inversely oriented TIMEs, as detailed below. The other is the successful spread of IncN plasmids carrying the TIME-COMP, as exemplified by the detection of these plasmids across multiple genera (i.e. *Citrobacter*, *Escherichia*, *Enterobacter*, *Klebsiella*, *Phytobacter* and *Salmonella*, Table S1).

**Table 1.** Summary of TIME-COMPs carrying ARGs in *Enterobacteriaceae*

Bounding TIME	Orientation of TIMEs	ARG(s)	Size (bp)	Flanking DRs	Replicon type(s)*	No. of replicons	References identifying TIME-COMP
TIME244	Inverted	*tet*(A)	3,414	AGCAA	IncN	114	This study
				TATAA	IncF	18	
				TACTT	IncR	1	
	Direct	*bla*_KPC-2_, Δ*bla*_TEM_	3,896	TATAT	Col(MGD2)	1	This study
				TGTTTA	IncQ1	6	
	Inverted	*qnrS1*	2,379	ATCCA	–	2	This study
IS*101*	Direct	*qnrD1*	1,956	AGCTA	Col3M/ColRNAI	1	Gomi and Adachi [12]
	Inverted	*bla*_KPC-2_	11,421	ATTCT	IncX3	1	This study
MITESen1	Direct	*qnrS1*	1,894	TAATA	IncX1	1	This study
	Direct	*floR*	4,800	TAATA	IncX1	1	This study
	Direct	*qnrD1*	1,259†	TATAT	repE(pEh60-7)	1	This study
IMU	Inverted	*bla*_GES-5_	2,160	-‡	IncQ	1	Poirel *et al*. [9]
	Inverted	*bla*_GES-5_, *aacA4*	2,799	-‡	IncQ	1	Pedersen *et al*. [11]

* When multiple replicons are present, the major replicon type is indicated.

† This TIME-COMP is interrupted by IS*26* in the original plasmid, and the size indicated here corresponds to that of the uninterrupted version.

‡ Although the IMU-bounded TIME-COMPs are not flanked by direct repeats in the original plasmids, the previous study reported that the IMU-bounded TIME-COMP carrying *bla*_GES-5_ was mobilized by a transposase provided in *trans* [9].

### Putative resolution sites in TIMEs

**Fig. 4.**
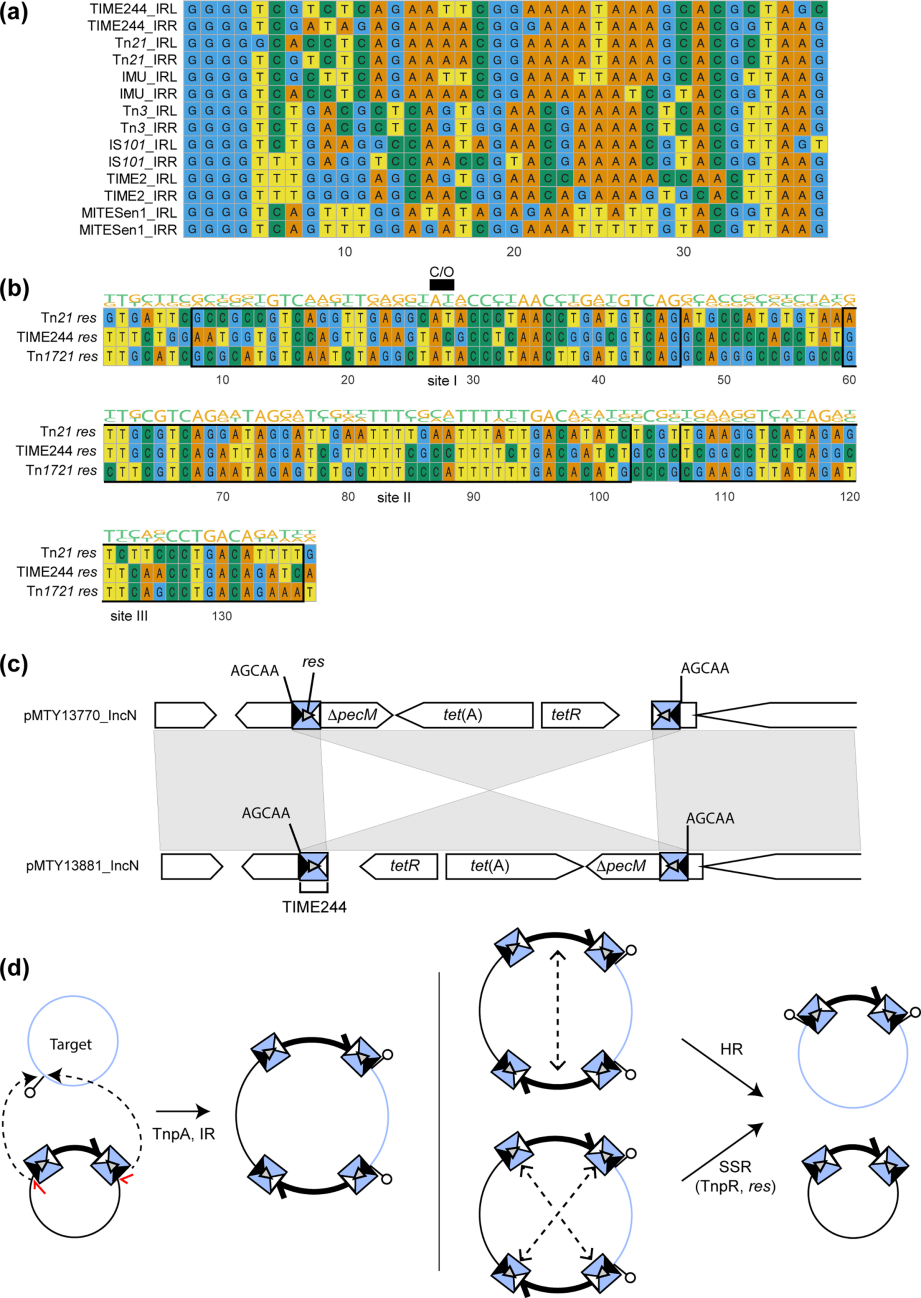
(**a**) Multiple alignment of IRs of TIMEs, Tn*3* and Tn*21*. The alignment was visualized using the Bioconductor package ggmsa [46]. (**b**) Multiple alignment of *res* sites of Tn*21*, TIME244 and Tn*1721*. The definition of subsites follows a previous study [38]. See Table S2 for the sequences of TIME244, IMU, IS*101* and MITESen1. The remaining sequences are derived from the following accession numbers: Tn*21*, AF071413; Tn*3*, HM749966; TIME2, KJ920398; and Tn*1721*, X61367. Conservation level is indicated over the alignment by sequence logos in ggmsa. (**c**) Two frequent forms of TIME244-bounded TIME-COMPs carrying *tet*(A) inserted in the same location. The genetic contexts in two IncN plasmids (pMTY13770_IncN: LC720959 and pMTY13881_IncN: LC720960) are shown as examples [47]. (**d**) Deduced transposition pathways of TIME-COMPs bounded by inversely oriented TIME244 copies. Cointegrate formation (left) is mediated by TnpA and IRs, followed by replication. Cointegrate resolution (right) is mediated by RecA-dependent homologous recombination (HR) between duplicated TIME-COMPs or by TnpR/*res*-dependent site-specific recombination (SSR). SSR can occur between either pair of the directly oriented TIME244 copies. Black circles indicate donor replicons, whereas light blue circles indicate target replicons. Filled and open triangles indicate the left and right terminal IRs of TIME244, respectively. Red arrows indicate nicking sites (3′ ends of TIME-COMP) in the donor molecule. Solid arrows indicate transition steps between structures. Dashed arrows connecting two regions indicate strand-nicking and transfer events between those regions, involved in cointegrate formation or resolution.

All TIMEs forming the TIME-COMP structures identified in the present study (i.e. TIME244, IS*101* and MITESen1) carry IRs related to Tn*3*-family transposons (Fig. 4a). IRs of TIME244 and the previously reported IMU show more similarities to IRs of Tn*21* than to those of Tn*3*, IS*101*, MITESen1 and *Pseudomonas*-derived TIME2, which is not associated with ARGs [7]. Thus, TIME244 and IMU seem to have originated from the Tn*21* subgroup of the Tn*3* family [13]. To deduce whether TIME244 contains a *res* site, we conducted all-against-all SSEARCH using sequences of TIMEs, Tn*21 res*, Tn*1721 res* and Tn*3 res* [22]. A significant match (S-W>100) was detected for pairs of TIME244-Tn*21 res* (S-W=148), TIME244-Tn*1721 res* (S-W=152), Tn*21 res*-Tn*1721 res* (S-W=188) and IS*101*-Tn*3 res* (S-W=139). Fig. 4b shows alignment of the TIME244 internal sequence and the experimentally determined *res* sites of Tn*21* and Tn*1721* [38]. The *res* sites of the Tn*3* family consist of three subsites (site I, site II and site III), where TnpR dimers bind. Strand exchange takes place at the centre of site I (C/O in Fig. 4b) [13]. The TIME244 internal sequence shows similarity to both Tn*21 res* and Tn*1721 res* at all three subsites. Therefore, we speculate that TIME244 contains a functional *res* site of the Tn*21* subgroup. On the other hand, the internal sequences of IMU and MITESen1 did not show similarity to well-characterized *res* sites from either autonomous Tn*3*-family elements or other TIMEs (S-W<70), although this does not exclude the possibility that these TIMEs carry resolution sites that serve as targets of site-specific recombinase(s).

In principle, the functionality of *res* affects the stability of the TIME-COMP structures. When *res* sites are directly oriented, the segment between two *res* sites can be deleted in the presence of TnpR. According to studies on autonomous Tn*3*-family elements, when *res* sites are inversely oriented, the segment between the two *res* sites can be inverted by TnpR *in vivo* [39,40], likely because knotted substrates carrying inversely oriented *res* sites, which are efficient substrates for TnpR [41], are produced *in vivo*. Among 142 replicons containing TIME244-bounded TIME-COMPs, only 7 replicons contain directly oriented TIME244 copies, while 135 replicons contain inversely oriented TIME244 copies (Table S1). This orientation bias is consistent with the idea that TIME-COMPs carrying directly repeated TIME244 copies are structurally unstable due to the intrinsic resolution activity in the presence of Tn*21*-related TnpR.

The most prevalent TIME244-bounded TIME-COMP was that carrying *tet*(A). This TIME-COMP carries TIME244 copies in inverse orientation. The TIME-COMPs carrying *tet*(A) in the 114 replicons with DRs of AGCAA are classified into those with an inverted internal structure (*n*=52) and those without (*n*=62) (Figs 1a and 4c). This inversion could be explained by TnpR-mediated site-specific recombination at the *res* sites. Because TIME244 contains a putative *res* site, the transposition of TIME244-bounded TIME-COMPs with inversely oriented TIME244 copies should follow a two-step process: cointegrate formation and cointegrate resolution in the presence of Tn*21*-related transposons (Fig. 4d). Cointegrate resolution can occur by RecA-dependent homologous recombination or site-specific recombination involving Tn*21*-related TnpR and *res* sites.

### Prevalence of TIMEs in RefSeq *Enterobacteriaceae* genomes

**Fig. 5.**
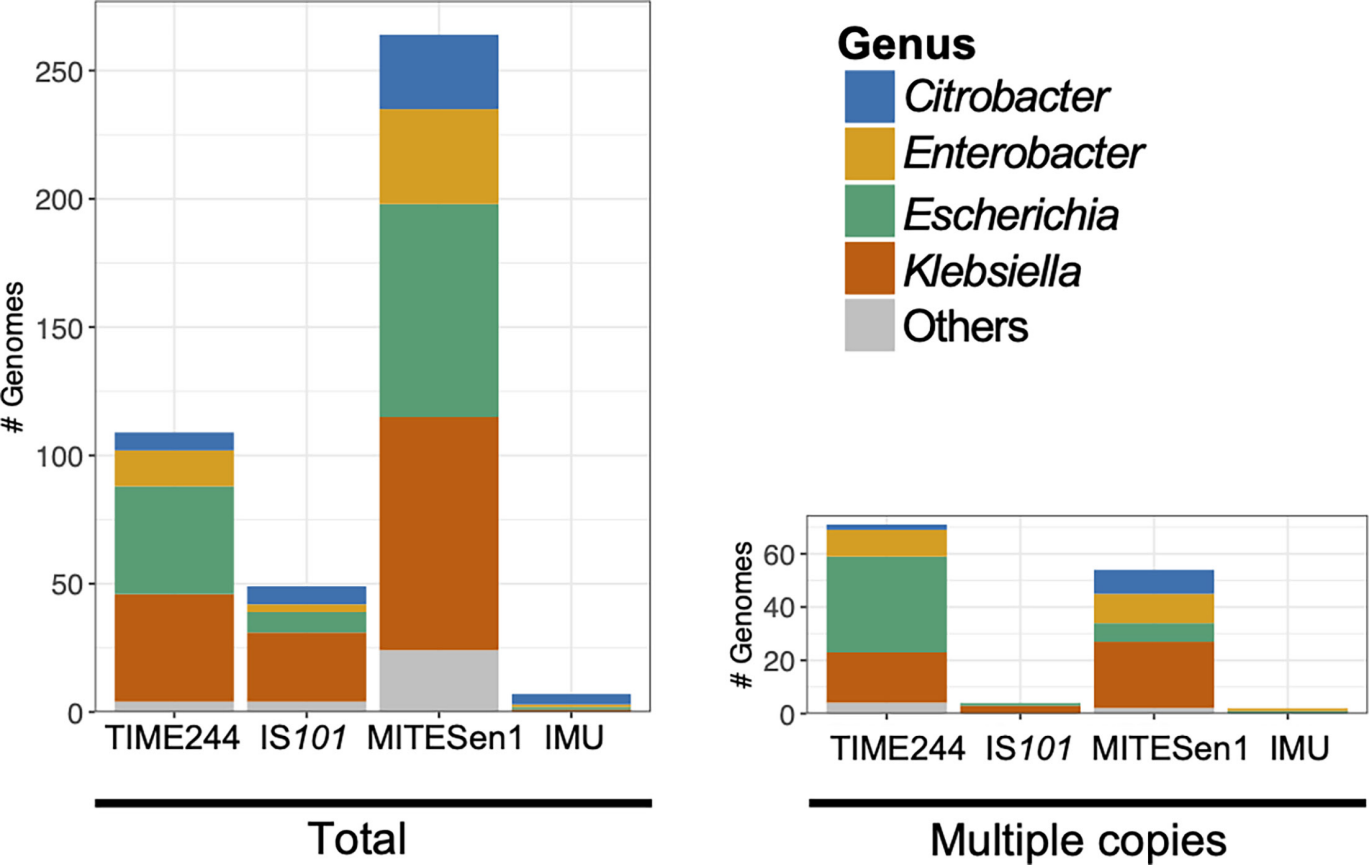
Number of RefSeq Enterobacteriaceae genomes carrying TIMEs (TIME244, IS*101*, MITESen1 and IMU) among the downloaded genomes (*n*=11,572). ‘Total’ indicates the total number of genomes carrying each TIME. ‘Multiple copies’ indicates the number of genomes with a replicon(s) carrying multiple copies of each TIME. Genera detected in >10 genomes are shown in different colours, while genera detected in ≤10 genomes are grouped as ‘Others’ and shown in grey. See also Table S3 for information on individual genomes and Table S4 for the numerical values presented in this figure.

The NCBI core_nt database, which we used in the online blastn analysis, contains a mixture of complete genomes and sequences that are not derived from complete genomes (e.g. plasmid sequences without the host chromosomal sequences). This prevents the accurate estimation of the prevalence of TIMEs associated with ARGs (i.e. TIME244, IS*101* and MITESen1, which were identified in this study within novel TIME-COMPs, as well as the previously reported IMU) in *Enterobacteriaceae* genomes. Thus, to circumvent this problem, we downloaded RefSeq complete *Enterobacteriaceae* genomes (*n*=11,572) and detected these TIMEs in the retrieved genomes. The frequency of genomes carrying each TIME was as follows: TIME244 (*n*=109, 0.94%), IS*101* (*n*=49, 0.42%), MITESen1 (*n*=264, 2.28%) and IMU (*n*=7, 0.06%) (Fig. 5 and Table S3). The frequency of genomes with a replicon(s) carrying multiple copies of each TIME was as follows: TIME244 (*n*=71, 0.61%), IS*101* (*n*=4, 0.03%), MITESen1 (*n*=54, 0.47%) and IMU (*n*=2, 0.02%). These TIMEs were mainly detected in clinically important genera, namely *Klebsiella*, *Escherichia*, *Enterobacter* and *Citrobacter*. However, these taxa are more likely to be sequenced due to their clinical importance, meaning the overall prevalence and burden of TIMEs in these genera may be inflated. Moreover, environmental or commensal bacteria, which are sequenced less frequently than clinical isolates, may harbour comparable TIME burdens. For example, the three *Leclercia* genomes carrying MITESen1 in our dataset were isolated from diverse sources (i.e. a clinical sample, a drain in a housekeeping closet in a hospital and pig feed) [42,44]. Thus, care should be taken to interpret the results. In total, 408 genomes (3.53%) harboured at least one of these TIMEs, indicating that these elements, while rare, are present at a detectable frequency. In replicons carrying at least one TIME, 444 (97.16%) were plasmids, and 13 (2.84%) were chromosomes.

### Study limitations

This study has limitations. First, the self-against-self blastn approach, which allows *de novo* identification of MITEs that form TIME-COMP-like structures, was performed only on small plasmids (≤20 kbp). Thus, novel MITEs potentially present in other types of replicons, such as larger plasmids and chromosomes, might have been missed. Specifically, there were 16,148 *Enterobacteriaceae* plasmids meeting criteria (i), (ii) and (iii) in the ‘Plasmid sequences’ section; however, only 1,007 (6.24%) small plasmids (≤20 kbp) were targeted in our *de novo* identification analysis, leaving larger plasmids, including 1,872 (11.59%) in the 20–50 kbp range, unexamined. Performing the self-against-self blastn analysis also on larger replicons may enable the identification of more MITE elements; however, this approach is unwieldy because it will return exponentially more blastn hits, which makes manual curation of the results (e.g. confirmation of DRs at the ends) almost impossible. A direction for future work would be to develop software for the automated detection of TIME-COMP-like structures. Moreover, there may be a prior literature bias in MITE discovery, which may have historically favoured small plasmids due to the ease of sequencing. This bias may also impact the broader database searches, potentially underrepresenting MITEs unique to larger plasmids or chromosomes. A second limitation of this study is that we analysed only the genomes of *Enterobacteriaceae*. Although rare, there seem to be TIME-COMP-like structures containing ARGs in other bacteria as well, such as *Acinetobacter* spp. [45]. Extending the approach employed in the present study to other bacteria may uncover additional TIME-COMP-like elements. Finally, while the transposition of certain TIME-COMPs was previously experimentally verified [7,9], experimental validation of the TIME-COMPs identified in the present study (especially those bounded by TIME244, which could be tested by providing a Tn*21*-related transposase in *trans*) would be a highly valuable next step to complement our bioinformatic findings.

## Conclusions

The present study identified multiple previously unreported TIME-COMPs containing ARGs. These novel TIME-COMPs are bounded by three types of TIMEs (i.e. TIME244, IS*101* and MITESen1). The sequences of these three TIMEs and also IMU are divergent and seem to have independently emerged from distinct transposons of the Tn*3* family. This study revealed that TIMEs contribute to the intracellular mobilization of ARGs and highlights the importance of taking these elements into account when analysing the genetic contexts of ARGs. Accurate identification of not only autonomous MGEs but also non-autonomous MGEs, including TIMEs, will enable us to capture the mobility landscape of ARGs more comprehensively in genomic surveillance.

## Supplementary material

10.1099/mgen.0.001790 Supplementary Material 1.

10.1099/mgen.0.001790 Supplementary Material 2.

## Abbreviations

ARGs: antibiotic resistance genes
DRs: direct repeats
IMU: integron mobilization unit
IRs: inverted repeats
ISs: insertion sequences
MDR: multidrug resistance
MGEs: mobile genetic elements
MITEs: miniature inverted-repeat transposable elements
S-W: Smith–Waterman scores
TEs: transposable elements
TIMEs: Tn*3*-derived inverted-repeat miniature elements
